# Is Consumption Ever Contagious, or Communicated by One Person to Another, in Any Manner?

**Published:** 1864-07

**Authors:** W. M. Cornell

**Affiliations:** Philadelphia, Penn.


### CORRESPONDENCE.

### IS CONSUMPTION EVER CONTAGIOUS, OR COMMUNICATED BY ONE PERSON TO ANOTHER, IN ANY MANNER?

BY W. M. CORNELL, M. D., LL. D., PHILADELPHIA, PENN

Dr. H. S. Bowditch, in the *Boston Medical and Surgical Journal*, Vol. lxx. No. 17, has handled this subject in an able manner, as he does all subjects that he attempts. Dr. B. has had much experience in this disease, and his observations are important to the profession. Still, I think he has arrived at erroneous conclusions respecting the communication of consumption from one person to another. I do not say it is *contagious;* because, strictly speaking, but few diseases are so; that is, communicated by simple *touch*, which is the real derivation of *contagious;* but, that this disease is communicated from one person to another in some way, I have not a doubt.

In a small work which was published in 1850, on "Consumption Treated," I said, "consumption is *contagious*. I mean by this, that it can be taken by one person from another. The ancient Romans so believed, and hence burnt the beds, clothes, furniture, and books of those who had died of this disease." Upon this point, I will add only one quotation from Dr. B.'s article, which he seems to have gathered from what he calls "the learned work of Dr. Young." In a note, Dr. B. says, "Practical and Historical Treatise on Consumptive Diseases, by Thomas Young, M. D., F. M. S., London, 1815."

Dr. B. continues, "It would seem from Dr. Young's statements, that all the chief authors who, previously to the last quarter of the last century, treated of the subject, were full believers in the contagiousness of consumption." He then says, "Aristotle, among the Greeks, Galen, Morton, of London, 1619; Phinius, 1728; Morganni, 1761, and Wothering in 1775—all these, and, indeed, every other writer of note, believed consumption was communicated from one person to another."

Now, all the use I wish to make of these writers, and of the general opinion held by them is this, old maxims and general opinions of the whole medical profession are usually correct. If it were so, in this case, then Dr. B.'s conclusion, that consumption is not communicated from one person to another, is not correct. I like the Doctor's caution. I am aware that it may be truthfully said, in reply to this remark, that the whole profession have often been wrong. This is true. *They* were wrong, and Jenner right, upon vaccination. *They* were wrong, and Harvey right, upon the circulation. *They* were all wrong, in this country, and abroad who followed *Rush*, as to his notions of venesections. whom a late eminent English physician says, "was peculiarly blood-thirsty." Many other cases of a parallel character might be given, but they are not necessary.

My reply to these cases is, they have not remained under these erroneous views for centuries, as the whole profession did respecting consumption, if that were an error. The profession soon acknowledged Jenner to be right; they soon adopted Harvey's plan of the circulation, and they have now almost to a man forsaken the blood-thirsty practice of Rush; and the presumption is, that the old medical writers would much earlier have abandoned their views of the contagiousness of consumption had not those views been correct. At least, I am satisfied that they were.

A single word may be said here upon Dr. B.'s remarks, as follows: "Only two writers of note and of undoubtedly strong minds, and these Americans, can be found to defend the proposition of contagiousness.—These two writers are nearly at the two extremities of the period named under consideration, viz: Benjamin Rush, 1793, and Drake, 1854."

*I* claim to have maintained this opinion, and published it in 1850.—But, I may not be a "writer of note, nor of a strong mind." I am far from attributing any intentional neglect to Dr. B. in this case, for two reasons; the first is, he is not *such* a man, but of liberal views and always above small things; and the second is, he reported favorably as a committee upon inhaling and otherwise using a powder of Nitrate Argent et Podophyllum by me, which report was, and still is, referred to in the *Dispensatory*, mentioning me by name, under the article *Argentum*. So that I

am satisfied to believe that it was a mere oversight in Dr. B. in not giving me credit here, as formerly.

One remark may be made respecting the writers of late, referred to by Dr. B., who maintain that consumption is *not* contagious; it is this, most of them add, "it is better not to sleep with a consumptive person." Why this caution, if there is no danger? Who would caution a man not to fly off to the moon? Does not such caution manifest a little want of confidence in the expressed opinion? One would certainly think so.

One other remark in passing, which is, old women and old nurses generally believe consumption to be contagious. I hear them express this opinion every day. It was expressed by one yesterday, whose wife lately died of the disease, and whose surviving husband now seems laboring under the same. It may be said, this is but an old woman's opinion, and is of no more value in the argument, than "Mrs. Winslow's Soothing Syrup" would be in this disease. I know all that, and I also know that old women first used, and from their first using, many medicines, they were adopted by the profession, and *thus* found their way to being officinal. Take, for instance, the best parturient we have, called by them *muttercorn;* by us *ergot.* This was long used by the German old mothers, before we used it.

I here add the same remarks as before, what everybody says is true; or generally has a foundation in fact. We find this verified respecting nine-tenths ot the old surgeon's adages.

One preliminary remark, and then I have some *facts* to adduce. I am, as you know, a *specialist*, and one of my *specialties* has long been *Phthisis.* Upon this I have written, and in treating it, as in one other specialty, I have had some patients from abroad. I am not purposing to discuss *specialties* here; but I do mean to do it sometime, as I am sure half the profession are wrong upon the subject.

A young man came to me as a *specialist*, with phthisis. I inquired, were either of your parents consumptive? The reply was, *no.* Were your grand-parents? No. They both lived to old age. The patient added, "my wife died of consumption, and I was with her, lived with and lodged with her, and my mother says I took the consumption from her." Now, here was a case in point. He was evidently going in consumption, and I could not learn that any of his near blood relatives had died of this disease. I think, with his mother, that he took it from his wife; and still, I believe wives oftener take it from their husbands. I have a case nearer home. I belonged to a consumptive family. My father, mother and an only brother, all died of phthisis; the latter before he was thirty years old. He married into a healthy family, no member of which, certainly for two generations, both on the father's and mother's side, had died of consumption. My brother had the true tuberculous consumption. His wife, always healthy and robust, nursed him, lodged with him to the last, and died of the same disease within two years of my brother. She certainly had no *hereditary* taint. How do you account for it? I believe she took the disease from her husband.

I have another case in point. A young man of my acquaintance, of a consumptive family, married a young lady in perfect health, who belonged to a family not one of which had died of consumption for many years. Her grand-father on each side and grand-mother and parents, were all healthy, and her brothers and sisters. Her husband sickened and died of phthisis. She lived with him, nursed and slept with him to the last. I was not her husband's medical attendant; but within a year from the time of his death, his widow came to me for medical advice. I found her much below the standard of health, in her father's family, with considerable emaciation, slight "*hacking cough*," as she called it, and all the well known symptoms of consumption. She said, "I am very much as my husband was when he was first taken sick, and do you think I could have taken the disease from him?" She died within two years. There is a large family of her brothers and sisters, and every one of them enjoy perfect health, and the whole family connection is as free from any tubercular taint as I have ever known in any family.

Now, I do not know how to account for these things unless consumption is contagious or infectious. I believe it is, and that the ancient physicians were right upon this subject, and that facts in my practice for twenty years fully harmonize with this belief.

I beg leave here to quote a page from my little work upon this subject, alluded to above, assuring my readers that my views have undergone no change upon this subject during the fourteen years that have elapsed since it was published, namely: "If a husband or wife has this disease, and the healthy companion continues to lodge with the sick one, does the healthy one escape the disease? Many cases of this kind I have known. The same is true of a sister, who takes the nursing of, and sleeps with a consumptive sister. I know it seems hard to the sick that a companion, or a near friend, should be afraid (as it is termed) to lodge with them. But it is the first law of nature to preserve one's self. If the sick *must suffer*

unless the healthy are thus exposed, the case would be different. But such is not the fact. They can have all proper and necessary attention, without the health of a friend being thus endangered. This breathing the air, as it issues from a decayed or decaying lung, and imbibing the perspiration from a consumptive body, is at the risk of health and life. Who has not seen a whole family of sisters, living together, and nursing each other, successively follow one another with fearful strides, to the dark and silent resting-place, while the one or two who had married early and had removed from the parental mansion, have escaped? I have seen many such cases."

I might quote still more, all going to show that I had written upon this subject, though not recollected by my friend Dr. B.

And now, Sir, may I call your attention to one other point not exactly connected with the one now under discussion, yet still relating to consumption. In a little book which I published in 1848, I spoke of the use of *alcohol* in consumption, and of preferring *to send consumptive patients North* instead of South. I had never seen either of these ideas in print, nor heard them expressed by any one, previous to that time; and I know that many physicians who now embrace both, *then* laughed at them.—If you are aware of their having been broached previous to 1848, tell me *where* I can find it.

